# Microbial Adhesion to Dental Polymers for Conventional, Computer-Aided Subtractive and Additive Manufacturing: A Comparative In Vitro Study

**DOI:** 10.3390/jfb13020042

**Published:** 2022-04-11

**Authors:** Sergey Arutyunov, Levon Kirakosyan, Lubov Dubova, Yaser Kharakh, Nikolay Malginov, Gadzhi Akhmedov, Viktor Tsarev

**Affiliations:** 1 Propaedeutics of Dental Diseases Department, A.I. Yevdokimov Moscow State University of Medicine and Dentistry, 127473 Moscow, Russia; sd.arutyunov@mail.ru (S.A.); dr.lkirakosyan@gmail.com (L.K.); 2 Orthopedic Dentistry Department, A.I. Yevdokimov Moscow State University of Medicine and Dentistry, 127473 Moscow, Russia; dubova.l@gmail.com; 3 Prosthodontics Technology Department, A.I. Yevdokimov Moscow State University of Medicine and Dentistry, 127473 Moscow, Russia; malginov_nn@mail.ru; 4 Surgical Dentistry Department, A.I. Yevdokimov Moscow State University of Medicine and Dentistry, 127473 Moscow, Russia; gahmedov@mail.ru; 5 Microbiology, Virology, Immunology Department, A.I. Yevdokimov Moscow State University of Medicine and Dentistry, 127473 Moscow, Russia; nikola777@rambler.ru

**Keywords:** microbiology, dentistry, prosthodontics, dental prosthesis, technology, dental, pathology, clinical, acrylic resins, bacterial load, clinical reasoning

## Abstract

Modern structural materials are represented by a variety of polymer materials used for dental patients’ rehabilitation. They differ not only in physico-chemical properties, but also in microbiological properties, which is one of the reasons why these materials are chosen. The study focused on the microbial adhesion of clinical isolates of normal (5 types), periodontopathogenic (2 types), and fungal (2 types) microbiotas to various materials based on polymethylmethacrylate (PMMA) intended for traditional (cold-cured and hot-cured polymers), computer-aided subtractive and additive manufacturing. A comparative analysis was carried out on the studied samples of polymer materials according to the microorganisms’ adhesion index (AI). The lowest level of microorganisms’ AI of the three types of microbiotas was determined in relation to materials for additive manufacturing. The AI of hot-cured polymers, as well as materials for subtractive manufacturing, corresponded to the average level. The highest level of microorganisms’ adhesion was found in cold-cured polymers. Significant differences in AI for materials of the same technological production type (different manufacturers) were also determined. The tendency of significant differences in the indicators of the microorganisms’ adhesion level for the studied polymer materials on the basis of the type of production technology was determined.

## 1. Introduction

The rapid development of digital technologies for the production of polymer dentures (computer-aided subtractive and additive manufacturing) has led to the expansion of their application possibilities and increased their importance.

For example, in cases of patients’ rehabilitation with malocclusion or partial edentulism with overextended toothless gaps, especially when complicated by temporomandibular disorders, the importance of dynamic control of new occlusive relationships often arises. In this regard, long-term provisional restorations are used [1]. Equally significant is the usage of temporary prostheses in the rehabilitation of patients with dental implants for the period of their osseointegration [2,3].

While choosing structural materials for temporary use during patients’ dental rehabilitation, it is necessary to be guided not only by their strength and aesthetic characteristics, but also by the peculiarities of the materials’ interaction with the oral cavity microbiocenosis [4]. Nowadays, the most common materials used in almost every field of dentistry are polymers based on polymethylmethacrylate (PMMA). Over time, not only does a biofilm form on the surface of the material, but microorganisms also penetrate into the underlying layers of the polymer material [5]. At the same time, there is a sufficient variety of PMMA materials, which differ based on the chemical additives included. Such additives are necessary not only to achieve certain physico-chemical and aesthetic properties, but also to ensure the polymerization reaction. Therefore, the polymerization of materials is closely related to the conditions for manufacturing temporary structures (clinical or laboratory), as well as the method of their production: conventional, computer-aided subtractive or additive [6,7]. The microbiological aspect in relation to polymer materials is quite an urgent issue, which is confirmed by the active study of existing materials [8], as well as the search for new materials with antibacterial properties [9,10]. It is known that *Streptococcus viridans*, *Actinomycetales*, *Veillonellaceae*, and *Corynebacterium*, which represent the normal microbiota, play a leading role in this biotope in terms of the oral cavity colonization frequency and quantitative representation [11,12]. At the same time, a number of species are regarded as periodontopathogenic. It is known that they can quickly colonize the biofilm on the surface of the prosthesis and negatively affect the condition of the oral cavity mucous membrane, teeth and periodontal condition, as well as the prosthetic structure itself [13,14].

According to the works of recent years, the fungal microbiota represented by yeast fungi of the genus *Candida* also play an equally important role. It can cause not only various local diseases, but also systemic ones (stomatitis, precancerous lesions and dysplasia of the oral mucosa, oral cancer, systemic candidiasis, etc.) [15,16,17,18].

The aim of this study is to assess the adhesion of representatives of normal, periodontopathogenic, as well as fungal microbiotas (yeast fungi of the genus *Candida*) to various types of polymer materials used in the manufacturing of temporary fixed dentures using different production technologies.

Based on the purpose of the study, the following null hypotheses were determined:

- H_0_1: There are no differences in the values of the adhesion index of normal microbiota to the materials of the studied groups;
- H_0_2: There are no differences in the values of the adhesion index of periodontopathogenic microbiota to the materials of the studied groups;
- H_0_3: There are no differences in the values of the adhesion index of fungal microbiota to the materials of the studied groups.

## 2. Materials and Methods

### 2.1. Study Design

#### 2.1.1. General Information

The design of this study was planned as a comparative analysis. We studied the adhesion of three types of microbiotas to nine polymer dental materials (Table 1).

A comparison between the study groups was carried out separately for each type of microbiota. The final data were taken as the general values of the adhesion index of all types of microorganisms constituting the corresponding type of microbiota: normal—5 species; periodontopathogenic—2 species; fungal—2 species. In this study, clinical isolates were used. The distribution of microbial species by microbiota types is shown in Table 2.

#### 2.1.2. Sample Size

The statistical method ANOVA one-way was used to test null hypotheses. The calculation of sample size was carried out in the G*Power program (v 3.1.9.6, Heinrich Heine Universität Düsseldorf, Dusseldorf, Germany), based on the following values set by us: significance (α)—0.05; power (1–β)—0.8; effect size (Cohen’s f)—0.25; and 9 study groups. In that regard, to test null hypotheses, it was necessary to produce 756 samples (28 samples in each group). However, while studying the normobiota, the number of samples was increased to 30 in order to distribute the samples equally among 5 strains. Since the calculation of the sample size did not take into account the number of studied components (strains) of the independent variable, a different number of samples were obtained in the studied groups. In total, the required number of polymer samples was 774 (Table 2).

### 2.2. Sample Making

All polymer samples had the shape of disks, with a diameter of 5 mm and a height of 1 mm. The places of supporting structures and excess acrylic (flash) on the samples were removed and subjected to post-processing with polishers of various abrasiveness in the following sequence: 9400.204.030, 9401.204.030, 9402.204.030 (Komet, Gebr. Brasseler GmbH & Co., KG, Lemgo, Germany).

#### 2.2.1. Computer-Aided Additive Manufacturing

In the ExoCad Gateway 3.0 program (Align Technology, Tempe, AZ, USA), a virtual sample was designed and then imported into Slicing software in *.STL format. Preparation of virtual models for additive manufacturing was carried out in accordance with the recommendations of materials manufacturers and equipment for three-dimensional printing. The samples’ print orientation was 90°. Parameters for the printing of the samples are presented in the Table 3.

#### 2.2.2. Computer-Aided Subtractive Manufacturing

Virtual models of samples were prepared in the program Modellier (ZirkonZahn GmbH, Gais, Italy). The production of polymer samples from materials Temp Basic A2-B2 (ZirkonZahn GmbH, Gais, Italy) and Re-Fine Acrylic A2 (Yamahachi Dental MFG., Co., Gamagori, Japan) was carried out on a computer-aided subtractive machine M5 (ZirkonZahn GmbH, Gais, Italy).

#### 2.2.3. Conventional Samples

The cold-cured and heat-cured polymer samples were made by the compression molding technique. Wax specimens were made by the computer-aided subtractive technique according to the virtual master model from the material Wax Disk Alpha (Yamahachi Dental MFG., Co., Gamagori, Japan). After packing wax blanks in flasks with gypsum and after wax elimination, resin was packed. After the packing of heat-cured polymer, polymerization in a water bath with a temperature regime in accordance with the manufacturer’s recommendations was performed.

### 2.3. Microbiological Techniques

The time period from the samples’ production to the implementation of the study of the microbiological part did not exceed 72 h. Before the in vitro experiment, the samples were cleaned in an ultrasonic cleaner for 15 min, after which they were treated with 70% ethyl alcohol.

To carry out the process of primary adhesion, samples of materials were placed in a test tube with 0.5% Oxoid Agar Bacteriological (Agar No. 1) (AM) (Thermo Fisher Scientific, Waltham, MA, USA) containing bacteria of a certain species (strain) at a known concentration—10^9^ CFU/mL for bacterial cultures and 10^8^ CFU/mL for yeast—according to the 0.5 McFarland standard [19].

The exposure was carried out under anaerobic conditions at a temperature of 37 °C for 40 min. After that, the samples were washed three times with a sterile isotonic sodium chloride solution (to remove non-adhering microbial cells) and placed in special cassettes with an AM with a volume of 2 mL. The cassettes were subjected to ultrasonic treatment with a power of 60 kHz in an ultrasonic cleaner for 10 min.

From each portion containing a sample of the test material, a suspension of microorganisms subjected to ultrasonic treatment was taken in 100 mcl of the AM and a sectoral seeding was performed on 5% Colombian blood agar with the addition of sterile defibrinated sheep blood (Himedia Labs, Mumbai, India—for bacterial cultures) or chromogenic medium (Himedia Labs, Mumbai, India—(for fungi of the genus *Candida*).

After quantitative seeding, bacteria were cultured under anaerobic conditions at a temperature of 37 °C for 7 days, and fungi were cultured at room temperature (25 °C) for 2 days.

The number of colonies grown on the samples’ surface after subjecting to the ultrasonic treatment was calculated using the Scan 500 device (Interscience, Saint-Nom-la-Bretèche, France). In this device, microbial contamination data computer processing was carried out in the Scan v. 5.0.2 program (Interscience, Saint-Nom-la-Bretèche, France).

The adhesion index (AI) was determined as the ratio of the decimal logarithm of the colony-forming unit (CFU) number, obtained after subjecting the studied samples to ultrasonic treatment, to the decimal logarithm of the CFU of the initial microbial suspension according to the formula

(1) $${\mathrm{AI}=\mathrm{lg}}_{10}\left(\frac{C_{1}}{C_{0}}\right),$$

where AI—adhesion index, C_0_—the CFU of the initial microbial suspension, and C_1_—CFU/mL after subjecting the samples to ultrasonic treatment.

### 2.4. Statistical Analysis

A statistical data analysis was performed using IBM SPSS Statistics 26 (IBM, Armonk, NY, USA). The ANOVA one-way method with a post hoc Tukey test was selected for samples with equal variances, the value of Levene’s test being *p* > 0.05. In other cases (Levene’s test *p* < 0.05), a Welch test was performed with a subsequent variance analysis and post hoc Games–Howell test.

## 3. Results

The variances in the studied groups of polymer samples with strains of normal, periodontopathogenic, and fungal microbiotas were not homogeneous according to the results of the Levene’s test (*p* < 0.05), while the value of the Welch test was significantly different (*p* < 0.05). This fact allowed to carry out a further analysis of variance, on the basis of which all null hypotheses about the absence of differences in the adhesion of the representatives of normal (H_0_1), periodontopathogenic (H_0_2), and mycotic microbiotas (H_0_3) to the studied polymer materials (*p* < 0.05) were rejected. The results obtained on the basis of the post hoc Games–Howell test are presented below.

### 3.1. Normal Microbiota

The values of cold-cured polymer materials (BC[C] and LT[C]) were significantly different from those of other materials (*p* < 0.05), while the values of their AI were the highest, 0.84 ± 0.04 and 0.81 ± 0.05, respectively.

The values of heat-cured polymer materials were also consistent; there were no significant differences between BH[H] and SM[H] (*p* > 0.05). The absence of significant differences in this group of materials was also determined in comparison with materials for computer-aided subtractive manufacturing (*p* > 0.05). Thus, the values of the AI of hot-cured polymers were 0.67 ± 0.05 for BH[H] and 0.65 ± 0.05 for SM[H], which were significantly higher than the values of the AI of the group of materials intended for additive manufacturing (*p* < 0.05).

In the group of materials for computer-aided subtractive manufacturing, significant differences were determined between TB[S] (AI = 0.70 ± 0.07) and RF[S] (AI = 0.64 ± 0.07) (*p* < 0.05). Significant intergroup differences were revealed in relation to groups of cold-cured materials and resins for computer-aided additive manufacturing (*p* < 0.05).

With respect to the material for computer-aided additive manufacturing of FP[A], both intragroup and intergroup significant differences in the AI index corresponding to the value of 0.55 ± 0.06 (*p* < 0.05) were determined. The remaining materials of this group, ND[A] and DS[A], had no significant differences only among themselves (*p* < 0.05). In comparison with other materials, the lowest adhesion of representatives of normal microbiota to ND[A] (AI = 0.48 ± 0.09) and DS[A] (AI = 0.44 ± 0.06) was determined.

**Figure 1:**
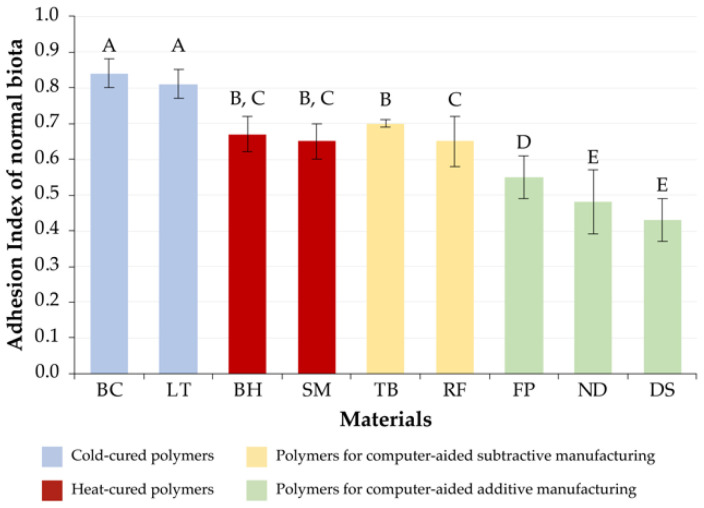
The adhesion index values of normal microbiota to the studied materials (270 samples). Same alphabetical letters above the bar graph indicate that there are no significant differences between the groups (*p* < 0.05).

The adhesion indices of normal microbiota to the studied materials are presented in Figure 1.

### 3.2. Periodontopathogenic Microbiota

The AI of BC[C] corresponded to 0.75 ± 0.03, and for LT[C] it was 0.73 ± 0.04. Significant differences were determined only for the AI of materials of other groups (*p* < 0.05), and their AI was the highest among the studied groups of materials.

The AI of the BH[H] (AI = 0.45 ± 0.04) turned out to be significantly different from that of all the studied materials, including the SM[H] (AI = 0.54 ± 0.09) (*p* < 0.05), with the exception of RF[S] (AI = 0.38 ± 0.16) (*p* > 0.05).

The AI of the TB[S] material corresponded to a value of 0.60 ± 0.02 and exceeded only that of the group of cold-cured polymers; significant differences were determined for all materials (*p* < 0.05). The AI of RF[S] (AI = 0.34 ± 0.05) differed significantly from those of cold-cured materials (BC[C], LT[C]), SM[H], and TB[S] (*p* < 0.05), while significant differences were not found in materials from the computer-aided additive manufacturing group (FP[A], ND[A] and DS[A]) and the hot-cured material (BH[H]).

The values of computer-aided materials had the lowest AI: FP[A] (AI = 0.34 ± 0.05), ND[A] (AI = 0.35 ± 0.04), and DS[A] (AI = 0.35 ± 0.02); significant differences were not found within the group (*p* > 0.05).

**Figure 2:**
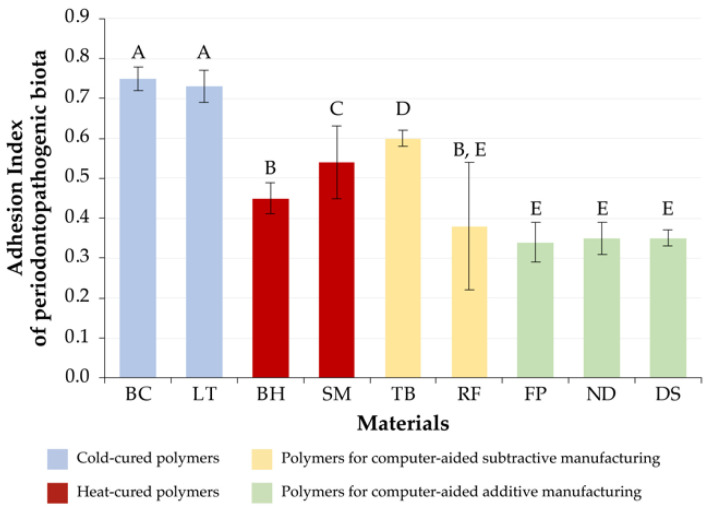
The adhesion index values of periodontopathogenic microbiota to the studied materials (252 samples). Same alphabetical letters above the bar graph indicate that there are no significant differences between the groups (*p* < 0.05).

The adhesion indices of periodontopathogenic microbiota to the studied materials are presented in Figure 2.

### 3.3. Fungal Microbiota

There were no significant differences between cold-cured BC[C] (AI = 0.69 ± 0.05) and LT[C] (AI = 0.69 ± 0.03) polymers (*p* > 0.05). As in the case of the periodontopathogenic microbiota within this group, we determined significant differences between the materials BH[H] (AI = 0.62 ± 0.03) and SM[H] (AI = 0.59 ± 0.02) (*p* < 0.05).

Moreover, significant intragroup differences were determined for materials of subtractive manufacturing (*p* < 0.05). At the same time, TB[S] (AI = 0.62 ± 0.03) had no significant differences from BH[H], and RF[S] (AI = 0.72 ± 0.05) from the cold-cured materials.

The AI of the materials of additive manufacturing significantly differed from that of the materials of other groups (*p* > 0.05). As a result of statistical intragroup comparison of indicators, there were not determined differences between FP[A] (AI = 0.43 ± 0.02) and ND[A] (AI = 0.41 ± 0.05) (*p* > 0.05). The values of DS[A] (AI = 0.34 ± 0.05) were significantly different from those of all the studied materials (*p* < 0.05), while its indicators of microorganisms’ adhesion were the lowest.

**Figure 3:**
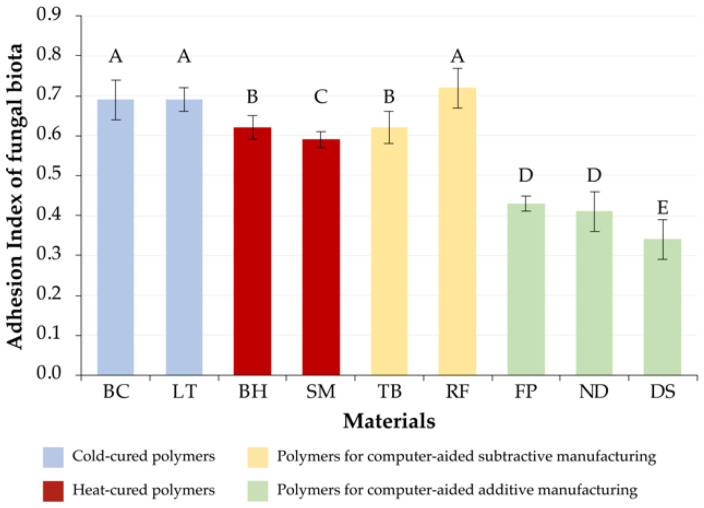
The adhesion index values of fungal microbiota to the studied materials (252 samples). Same alphabetical letters above the bar graph indicate that there are no significant differences between the groups (*p* < 0.05).

The adhesion indices of fungal microbiota to the studied materials are presented in Figure 3.

### 3.4. Summary Data

The materials were ranked based on the results of statistical analysis. The ranks were streamlined from the lowest to the highest, in accordance with the AI values obtained. In cases with no significant differences, the same ranks were assigned (Table 4).

As the result of the data presented in the table, the minimum AI (rank 1) was set for DS[A] material for all types of microbiotas, ND[A] material for normal and periodontopathogenic microbiotas, and FP[A] material for only periodontopathogenic microbiota. Samples of ND[A] and FP[A] materials for other types of microbiotas and samples of BH[H] for only periodontopathogenic microbiota also approached this group of materials in terms of relatively low adhesion (rank 2). The results obtained should be regarded as ranks of the high quality of these materials that prevents the adhesion of periodontopathogenic and fungal microbiotas. Low AI of normal microbiota, in our opinion, is also important, since it is known that the formation of the oral cavity mixed microbial biofilm occurs during the coagulation of periodontopathogenic bacteria with representatives of normal microbiota and yeast fungi.

## 4. Discussion

The rejection of the null hypotheses during the research allows us draw conclusions about the influence of polymer material manufacturing methods for temporary structures on the level of microorganisms’ adhesion to them.

This study is more focused on the comparison of manufacturing technologies of polymer materials and their impact on the materials’ microbiological properties.

In this research, we confined ourselves to studying only the adhesion level, since this parameter can be perceived as the cumulative result of the multifactor influence. Surface roughness and hydrophobicity (surface free energy) are considered the most important factors [20,21,22]. It is worth noting that a fundamental parameter is the materials’ chemical composition, a slight change in which affects both the microbiological characteristics [12,23] and the possibility of achieving the smoothest surface [24,25]. Studies have also revealed that the quality of additive manufacturing and the surface roughness of the samples are significantly affected by the printing parameters (print orientation, layer thickness, localization of supporting structures, etc.) [26,27,28,29]. Based on this, we manufactured samples that allowed us to achieve the most optimal surface quality possible.

It is worth mentioning that reducing roughness due to the final processing and post-processing with polishers is an effective way to restrain the formation of biofilm on a polymer structure [30]. However, the choice of material should be based on its initial roughness parameters, since the achievement of an adhesion-resistant surface can be resource intensive, as well as nondurable due to chemical and mechanical influences [31,32,33,34]. This fact is especially important in the case of long-term polymer structure usage, and therefore, in the present study, final processing and post-processing of the samples’ surface were not carried out.

Based on the results of a comparative analysis of polymer materials for different manufacturing techniques, M. Revilla-León et al. [35] discovered significant differences in surface roughness parameters between types of manufacturing. However, the lowest roughness parameters were found in the conventional manufacturing group, which contradicted our results. Nevertheless, according to their data, the FreePrint Temp material had significantly higher roughness parameters in comparison with NextDent C&B MFH, which correlated with the significant difference in adhesion indices of these polymer materials identified as a result of our study, with the exception of periodontopathogenic microbiota parameters.

Fiore A.D. et al. [36] studied the microbial adhesion of representatives of *S. mutans*, *L. salivarius*, and *C. albicans*. After 90 min of sample incubation, they observed the lowest adhesion of *S. mutans* and *C. albicans* to the samples of additive manufacturing, and of *L. salivarius* to the samples of subtractive manufacturing, which was comparable with the conclusions of our study.

Heat-cured resins for conventional manufacturing and polymer materials for computer-aided subtractive manufacturing are similar in nature and principles of polymerization. However, the manufacturing process of CAD/CAM blanks differs significantly in its conditions (high temperature and pressure), which reduces the level of residual monomer in the material and increases its strength characteristics [37,38]. It is thought that a convention degree of polymer materials significantly changes the activity of bacterial biofilm. This aspect has been shown in experiments with *S. mutans* [39] and *C. albicans* [40].

In a study by Al-Fouzan A.F. et al. [41] and Murat S. et al. [42], a higher significant difference in values of microorganisms’ adhesion to conventional polymer samples in comparison with samples of computer-aided subtractive manufacturing was revealed. In the course of our study, significant differences were revealed only for SM[H] and TB[S] materials, while the adhesion of fungal microbiota to conventional samples was less. This may be related to the consideration of materials of other manufacturers in our study and acceptance of a set of values of *C. albicans* and *C. krusei* as the final result.

According to A. Meirowitz et al.’s assessment of the adhesion of *C. albicans* to polymer material samples, the highest level of adhesion was reliably determined to the samples for additive technology, and the lowest to subtractive ones. The intermediate position is occupied by heat-cured and cold-cured samples [43].

A comparative study of heat-cured and cold-cured polymers by He X.Y. et al. [44] revealed a higher adhesion of *C. albicans*, *C. glabrata*, and *C. krusei* bacteria to cold-cured materials, which was consistent with the results of our study.

The considered composition of the microbiota is represented by the most significant representatives of each; however, it should be borne in mind that the spectrum of microorganisms in clinical conditions is wider, and therefore the possibility of supplementing the presented data in in vivo experiments is determined.

The design of the present study is focused on an isolated observation of the strains’ characteristics, which excludes any possible relationships of microorganisms (antagonism, competition, etc.). It is possible that as a result of the combined incubation of microorganisms on polymer samples, the level of their adhesion will be different. Therefore, the formation of a mixed biofilm may be the subject of a separate study.

The samples of materials for additive manufacturing presented in this study are made in the form of a simple geometric shape—a disk, which certainly affected the quality of the surface. It should be borne in mind that all products in dental practice have a complex geometry that complicates the optimization of the printing parameters, and, as a result, surface roughness increases. Thus, the shape of the samples examined for the level of microorganisms’ adhesion can influence the results.

The data obtained by us and presented in the scientific literature indicate the disunity and incompleteness of information regarding materials of computer-aided additive manufacturing. The need for further study of the properties and mechanisms of microorganism colonization of polymer materials remains relevant.

## 5. Conclusions

Within the limitations of this research, based on the findings of the present in vitro study, the following was concluded: depending on the type of microbiota, manufacturing technology, as well as the manufacturer of the material, the level of microorganisms’ adhesion to polymer materials significantly differs.

At the same time, the tendency of intergroup differences on the basis of the type of production technology was determined.

## Figures and Tables

**Table 1:** Characteristics of the studied polymer materials.

Material	Code	Manufacturer	Composition	Manufacturing Technology [Code]
Belakril-M HO Tempo, A2	BC	LTD “TD Vladmiva”, Belgorod, Russia	PMMA ^a^	Conventional cold-cured polymer [C]
Luxatemp Automix Plus, A2	LT	DMG Chemisch-Pharmazeutische, Fabrik GmbH, Hamburg, Germany	Glass filler in a matrix of multifunctional methacrylates; catalysts, stabilizersand additives. Free of methyl methacrylate and peroxides. Total filler volume: 44 w.% = 24 vol.% (0.02 to 2.5 μm) ^a^	
Belakril-M GO Tempo A2	BH	LTD “TD Vladmiva”, Belgorod, Russia	PMMA ^a^	Conventional heat-cured polymer [H]
Sinma-M, A2	SM	AO “Stoma”, Kharkiv, Ukraine	Acrylic fluorine-containing heat-polymerized resin of powder-liquid type ^a^	
Temp Basic, A2–B2	TB	ZirkonZahn GmbH, Gais, Italy	PMMA, 1% pigments ^b^	Computer-aided subtractive manufacturing [S]
Re-Fine Acrylic, A2	RF	Yamahachi Dental MFG., Co., Gamagori, Japan	PMMA with crosslinker and pigments ^b^	
FreePrint Temp 385, A2	FP	DETAX GmbH & Co. KG, Ettlingen, Germany	Liquid, light-curing (meth)acrylate-based onecomponent material ^b^	Computer-aided additive manufacturing [A]
NextDent C&B MFH, N2	ND	NextDent B.V., Soesterberg, Netherlands	Dimethacrylate-based resins with photo-initiator, and pigments ^b^	
Dental Sand, A1–A2	DS	Harz labs, Moscow, Russia	(Meth)acrylated oligomers, (meth)acrylated monomers, photo-initiator ^a^	

^a^—information provided by the manufacturer in the product description or instruction; ^b^—information based on 510(k) Pre-market Notification of US Food and Drug Administration.

**Table 2:** Quantitative distribution of samples by group.

Microbiota	Clinical Isolates	Number of Samples (*n* = 774)								
		BC	LT	BH	SM	TB	RF	FP	ND	DS
Normal	*Streptococcus sanguinis*	6	6	6	6	6	6	6	6	6
	*Streptococcus intermedius*	6	6	6	6	6	6	6	6	6
	*Staphylococcus aureus*	6	6	6	6	6	6	6	6	6
	*Staphylococcus warnery*	6	6	6	6	6	6	6	6	6
	*Corynebacterium xerosis*	6	6	6	6	6	6	6	6	6
Periodontopathogenic	*Porphyromonas gingivalis*	14	14	14	14	14	14	14	14	14
	*Prevotella intermedia*	14	14	14	14	14	14	14	14	14
Fungal	*Candida albicans*	14	14	14	14	14	14	14	14	14
	*Candida krusei*	14	14	14	14	14	14	14	14	14

**Table 3:** Information about computer-aided additive manufacturing methods.

Manufacturing	Material		
	FP	ND	DS
Slicing software	Asiga Composer v. 1.1.7 (Asiga, Alexandria, Australia)	PreForm v. 3.23.0 (Formlabs, Somerville, MA, USA)	Chitubox PRO v. 1.1.0 (ChiTuBox, Shenzhen, China)
Device	MAX UV (Asiga, Alexandria, Australia)	Form 2 (Formlabs, Somerville, USA)	Mono X (Shenzhen Anycubic Technology Co., Ltd., Shenzhen, China)
Tech.	Digital light processing printing technology (DLP)	Stereolithography printing technology (SLA)	Digital light processing printing technology (DLP)
Specifications	Manufacturer’s recommendations		Lift speed: 100 mm/min Retract speed: 1.7 mm/s Wait before print: 4 s
Layer thickness	50 µm	50 µm	50 µm
Post-processing	Anycubic Wash & Cure 2.0 (IPA 70%, wash 3 min, UV 30 min)		

**Table 4:** The ranking of polymer materials by AI values (M ± SD) based on the significant differences (*p* < 0.05).

Rank	Microbiota		
	Normal	Periodontopathogenic	Fungal
1	DS[A] (0.43 ± 0.06) ND[A] (0.48 ± 0.09)	FP[A] (0.34 ± 0.05) DS[A] (0.35 ± 0.02) ND[A] (0.35 ± 0.04)	DS[A] (0.34 ± 0.05)
2	FP[A] (0.55 ± 0.06)	RF[S] (0.38 ± 0.16) BH[H] (0.45 ± 0.04)	ND[A] (0.41 ± 0.05) FP[A] (0.43 ± 0.02)
3	SM[H] (0.65 ± 0.05) RF[S] (0.65 ± 0.07) BH[H] (0.67 ± 0.05) TB[S] (0.70 ± 0.01)	SM[H] (0.54 ± 0.09)	SM[H] (0.59 ± 0.02)
4	LT[C] (0.81 ± 0.04) BC[C] (0.84 ± 0.04)	TB[S] (0.60 ± 0.02)	BH[H] (0.62 ± 0.03) TB[S] (0.62 ± 0.04)
5	-	LT[C] (0.73 ± 0.04) BC[C] (0.75 ± 0.03)	LT[C] (0.69 ± 0.03) BC[C] (0.69 ± 0.05) RF[S] (0.72 ± 0.05)

## Data Availability

The data presented in this study are available on request from the corresponding author.
